# Standing in the gap: The academic and professional divide between health administration and health policy

**DOI:** 10.3389/fpubh.2023.1147646

**Published:** 2023-03-16

**Authors:** Steven W. Howard, Michael A. Counte, Leah J. Vriesman, Harrison D. Brink

**Affiliations:** ^1^Health Services Administration, University of Alabama at Birmingham, Birmingham, AL, United States; ^2^Health Management and Policy, Saint Louis University, St. Louis, MO, United States; ^3^Health Policy and Management, University of California, Los Angeles, Los Angeles, CA, United States

**Keywords:** health administration education, health policy education, pedagogy, curricula, career path

## Introduction

There are diverse types of health care systems, policies and health care organizations existing in health care delivery arrangements across the world (1). Two distinct influential groups of professionals have emerged as a result. They are: 1. Macro-level, population-oriented heath policy planners and analysts (health policy professionals–HPP) and 2. More micro-level, institutionally-oriented managers and organizational leaders and executives (health management professionals–HMP). As the old idiom goes, “one can't see the forest for the trees.” Are HPP professionals at such “altitude” that they have a clear vision of the forest, but fail to understand how things are on the ground at the “tree” level? Are HMP professionals so close to daily operations that they see only their “tree” and fail to fully understand how they fit in the larger “forest” (or understand the externalities of their organization-level decisions on the overall community or nation)?

Given the influence of HPP professionals in health planning at every level, and the role of HMP professionals in leading health care organizations to successfully adapting to environmental policy changes, building a strong understanding of each other and greater coordination between these professions would be beneficial to health care organizations around the globe. This project contrasts educational structures and processes that influence HMP and HPP educational formation and perspectives on major health issues. The issues and discussion in this report can facilitate building bridges across these two vital groups and greater collaboration over the long-term.

## Overview/history of health management educational programs

Health care delivery organizations (hospitals in particular) have had a longstanding existence in the Middle East, East Asia, Western Europe, and North America. However, there were no formal educational programs in health management until the 1940's (e.g., Saint Louis University, the University of Chicago, the University of Michigan, and Northwestern University). They were originally graduate level Master of Health Administration (MHA) programs housed in medical schools, schools of public health or business. Continued growth in the sector has created many more resident and distance-based programs in health management at baccalaureate, masters, and doctoral levels. Coursework includes domains such as: health care systems and organization, organizational theory, healthcare financial management, strategic management and planning, leadership, quantitative methods, health law, and information systems. The instructional model was historically face-to-face, but in the last 10 years demand for convenience and access has evolved a mixed model with both face-to-face and distance-based elements growing rapidly across the world. However, their primary goal continues to be producing graduates who will help health care organizations improve their efficiency and effectiveness amidst a continually challenging environment. The primary focus of health management programs is largely restricted to the micro- or institutional-level of analysis (e.g., widespread utilization of cases that address how individual health care organizations such as hospitals, practice management organizations, and long term care facilities respond to challenges to their strategy and operations).

## Overview/history of health policy educational programs

The history of education for health policymakers has been somewhat different. While early U.S. programs were also first accredited in the 1940's, most were organized around schools of public health (2). HMP education debates had centered around whether healthcare management was at its core about managing a healthcare business venture or managing community health (with hospitals as just one component), and therefore whether HMP programs belonged in business schools, public health, allied health/health professions, medical schools or other homes (3). The HPP education programs had not had this philosophical struggle. From the beginning, HPP had an unambiguous fit within schools of public health (3). With greater consensus about its logical home, HPP programs (or Policy concentrations within programs) would place Policy students and faculty in closer contact with their colleagues in the other public health disciplines (epidemiology, biostatistics, environmental health, and health education/health promotion). These diverging backgrounds would contribute to prevalent chasms between HPP and HMP programs and their students. As the business and policy challenges of health care organizations and countries have changed over the decades, so HPP and HMP programs continued to change, as would their collegiate homes. The evolution of the allied health professions would also add complexity to the questions of where policy and management skills are needed, and therefore which students needed to build those competencies, and where HMP and HPP programs should reside within the university. By 2017, one third of HMP programs were in public health schools (alongside HPP programs), one-third in health professions/allied health schools, and the remainder in business schools and other settings (3).

## Systematic comparison of typical HPP/MPH vs. MHA/HMP programs and impacts on graduates

**Figure 1:**
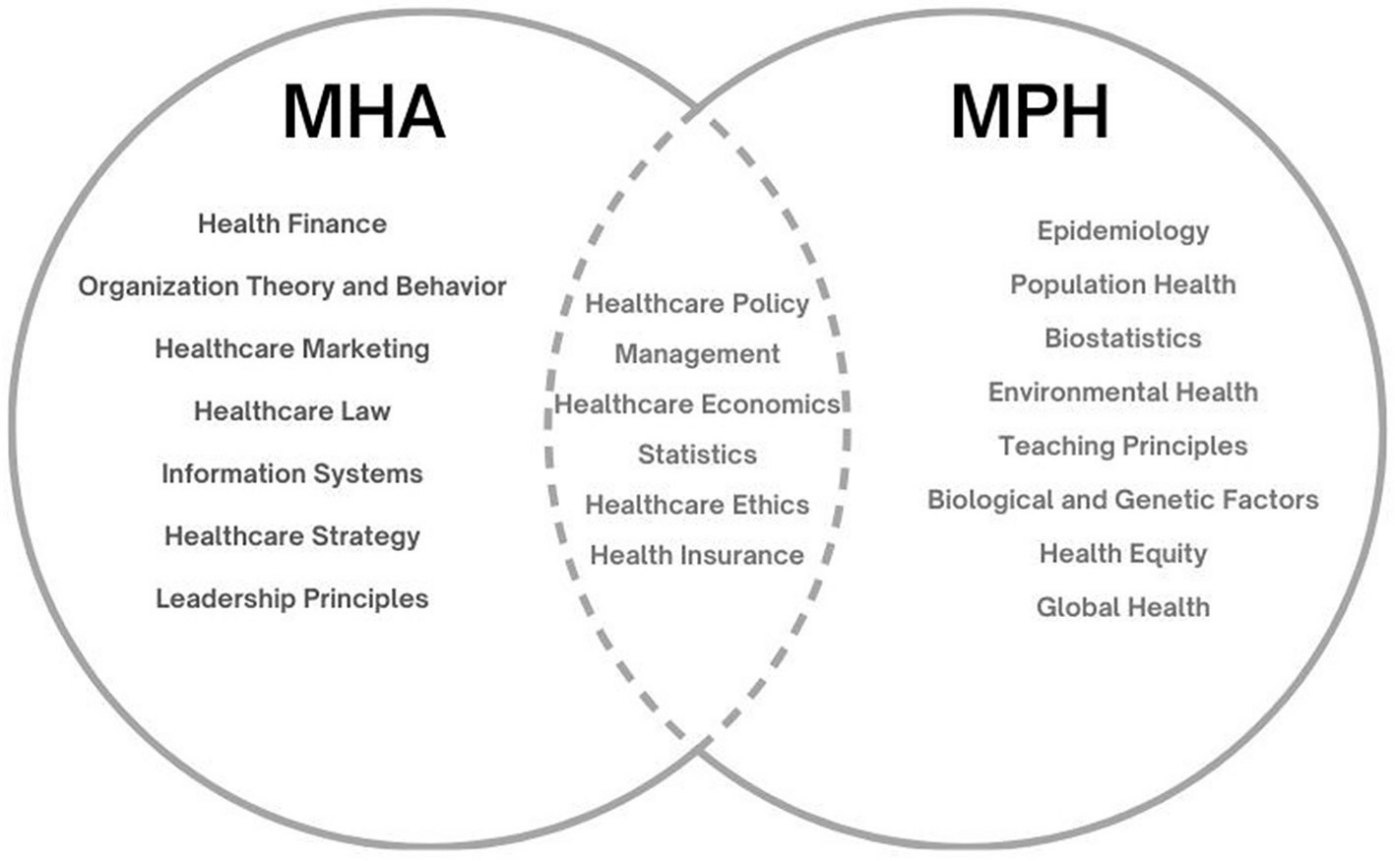
MHA and MPH program curriculum.

Although the increasing need for management-competencies within healthcare organizations has contributed to some divergence between HPP and HMP programs, some overlap exists between most programs' curricula (Figure 1).

We reviewed program curricula at 11 universities with leading HMP/MHA and HPP/MPH programs accredited by CAHME and CEPH, respectively (UCLA's MPH in Health Management had recently opted to discontinue CAHME accreditation, though it retained CEPH accreditation). Most commonly, both groups of students are required to take an introductory Health Policy course, Health Economics, and an Introductory Statistics/Quantitative Analysis class. Many programs also commonly required introductory Health Care Management, Ethics, and/or an Insurance/Reimbursement-related course. Beyond that shared coursework, curricula diverged significantly, with MHA/HMP students taking more traditionally business-oriented courses and MPH/HPP students studying more public health and health policy-related subjects.

According to those policy programs (or policy emphases) and other enrollment information, the most common initial jobs for HPP graduates are public health analyst, health policy analyst, and healthcare researcher; often for federal and state governments, institutes, think-tanks, and non-governmental organizations (NGOs). In addition, MPH in Policy grads can also pursue a number of positions that are often thought a closer match for MHA graduates (4, 5). To the extent health policy graduates pursue traditionally management/HMP jobs, they may be underprepared compared to MHA graduates also applying. Similarly, MHA/HMP graduates pursuing jobs with state or federal government, such as the Centers for Medicare and Medicaid Services (CMS), NGOs, etc. may be underprepared for the more community-based and public health-oriented aspects of those positions.

If MHA/HMP students interested in a more policy-oriented career path were able to gain additional competencies from the HPP/MPH side (Figure 1), would they be better prepared for success in their first jobs overall (especially now that population health is in demand within integrated health systems)? Likewise, would the HPP/MPH graduates have more success in the health care organizations job market if they gained management competencies reflected in the MHA/HMP side of the curriculum? (Figure 1).

## Changes needed

If policy and management programs could more effectively educate their students inter-professionally, what benefits could result? If MPH/HPP students learned more about the inner workings of the business of health care, would their state and federal health policies be more effective? Would they create a more harmonious relationship between the “governors and the governed”? Would they be more competitive for industry jobs if their career paths diverged from the traditional policy sphere? What benefits would accrue to HMP/MHA students from learning more from the health policy world? Management graduates could better understand macro-level issues that manifest at their organizational/micro-levels. Could they serve as better partners with policymakers in creating more practical, effective health policies, requiring fewer cycles of failure and policy revisions? Some students have expressed concern over an inadequate understanding of pertinent health policies such as the No Surprises Act, which may disproportionately impact the careers of HMP/MHA students. Incorporating detailed curriculum regarding health policies would allow students the opportunity to better understand the origin of these policies, as well as how they will influence their future careers. This knowledge may also improve future administrators' abilities to contribute to ongoing policy revisions and new policy creation. For example, the Pioneer ACO designs from the U.S. CMS had design flaws that deterred early adopters from remaining in the program long-term. Could better interprofessional understanding and planning have helped create a better design from the beginning rather than requiring a decade to improve the ACO model through trial and error at the organizational level? Similarly, could middle and upper-level administrators in hospitals and health systems be more effective, proactive advocates with their state and federal policymakers if they better understood how health policy is made, implemented, and amended? Today, most hospitals depend on their state & national hospital associations to advocate for them. Would we have more equitable and effective health policies if more of the impacted parties were health policy-literate? There is great opportunity for more win-win experiences between the health care sector and policymakers if HMP and HPP professionals better understand each other and collaborated more effectively.

## Conclusions

In this paper, we have contrasted the complementary roles of health care policy planners and health care managers. We have also compared the content of relevant educational programs, graduate knowledge, capabilities and competencies and their interdependence. In order for health care delivery systems to be effective and efficient, much more research on the interdependence of policy makers and administrators is needed. Questions include:

- What is the appropriate level of overlap needed to improve system and institutional decision making?
- What parties should be responsible for educational and professional improvement efforts (e.g., government, professional associations, etc.?).
- What do health services researchers need to study with respect to the implementation of relevant educational and professional development interventions (value-added)?

As a final conclusion, the authors reiterate that improved collaboration between policy makers and health care administrators is integral to bridging the gap and improving health care delivery.

## Author contributions

MC and SH conceived and drafted the majority of the text. LV contributed important content, particularly in the comparison and contrast, and Conclusion Sections. HB conducted the searches for curricula and job placement information and contributed substantively to the Changes and References Sections. All authors contributed to the article and approved the submitted version.

## References

[1] Counte M Ramirez B West D Aaronson W. The Global Healthcare Manager: Competencies, Concepts, and Skills. Chicago, IL: Health Administration Press (2018).

[2] Council on Education for Public Health (CEPH). Available online at: https://ceph.org/about/org-info/ (accessed December 22, 2022).

[3] Meacham M. Looking Back to Look Forward: AUPHA at 70. Chicago, IL: Health Administration Press (2018).

[4] HealthGrad.com. Why Earn a Health Policy and Management Degree. Available online at: https://www.healthgrad.com/healthcare/why-earn-health-policy-degree/ (accessed December 22, 2022).

[5] BestColleges. 5 Careers for a Master's in Health Policy Graduate. Available online at: https://www.bestcolleges.com/healthcare/careers/masters-health-policy/ (accessed December 22, 2022).

